# Alzheimer’s disease PSEN-2 N141I mutation reveals altered and shear-sensitive brain endothelial cell-like phenotype in human iPSC-derived models

**DOI:** 10.1186/s40478-025-02152-3

**Published:** 2025-11-11

**Authors:** Lily E. Takeuchi, Jennifer Lam, Craig A. Simmons

**Affiliations:** 1 Institute of Biomedical Engineering, University of Toronto, 164 College Street, Toronto, ON M5S 3G9 Canada; 2 Translational Biology and Engineering Program, Ted Rogers Centre for Heart Research, 661 University Ave, Toronto, ON M5G 1X8 Canada; 3 Department of Mechanical and Industrial Engineering, University of Toronto, 5 King’s College Road, Toronto, ON M5S 3GB Canada

**Keywords:** Alzheimer’s disease, Stem cells, Blood-brain barrier, Brain endothelial cells, Hypoperfusion, Microfluidics

## Abstract

Drug discovery efforts in neurological diseases, such as Alzheimer’s disease (AD), have had particularly poor outcomes due to the lack of models that recapitulate drug interactions at the cerebral vasculature. There is an unmet need to develop physiologically relevant models to study the impacts of blood flow-induced shear stress. In this work, we use a microfluidic platform to model the cerebral vasculature in AD using patient-derived brain endothelial-like cells (BECs). Induced pluripotent stem cells derived from a patient with familial AD (PSEN-2 N141I) and an unaffected control line were differentiated into BECs (AD2-BEC and fControl-BEC, respectively). BECs were exposed to static conditions or 12 dynes/cm^2^ of shear stress for 72 h prior to assessment of barrier permeability using fluorescent tracer assays, monocyte adhesion, and efflux transport function using receptor-inhibition assays. Upon shear conditioning, BECs demonstrated shear responsiveness through greater cell alignment in the direction of flow. AD2-BECs demonstrated reduced capacity for efflux transport by p-glycoprotein (P-gp), breast cancer resistant protein (BCRP), and multidrug resistant protein (MRP1) compared to controls (fControl-BECs, *p* = 0.0017, *p* = 0.0004, *p* = 0.0002, respectively). Upon application of shear conditioning, impairments to efflux transport in AD2-BECs were ameliorated. AD2-BECs also exhibited increased monocyte adhesion (2.2 ± 0.4-fold; *p* < 0.0001) which was further reduced by the application of shear stress in both lines. Taken together, these observations suggest the lack of shear stress exacerbates altered BEC phenotype in fAD. To our knowledge, we present the first in depth functional characterization of in vitro AD patient-derived BECs in both static and physiologically relevant shear conditions in which lack of shear reveals dysfunction of the cerebral endothelium in AD relevant to drug transport and immune cell trafficking.

**Supplementary Information:**

The online version contains supplementary material available at 10.1186/s40478-025-02152-3.

## Introduction

Alzheimer’s disease (AD) is the most common cause of irreversible dementia worldwide [1]. Nearly three decades ago, the amyloid hypothesis, which posits that the accumulation of amyloid beta (Aβ) is the primary driver of neurodegeneration, was first proposed [2–4]. Aβ is a ~ 4.5 kDa peptide derived from the processing of amyloid precursor protein (APP), and is the primary component of senile plaques in the brains of patients with AD. The identification of AD-penetrant gene variants in APP and its processing proteins, presenilin-1 (PSEN-1) and presenilin-2 (PSEN-2), provided groundwork towards the hypothesis. Since then, a plethora of evidence has been generated observing the induction of Aβ deposition and unravelling the mechanisms of neuronal toxicity, lending compelling support towards the central role of amyloid in AD pathogenesis [5–7].

More recently, the neurovasculature has emerged as a major player in AD pathology, leading to the proposal of vascular hypothesis of AD [8–10]. The vascular hypothesis posits that initial damage to cerebral vasculature (due to factors related to aging, lifestyle, and/or associated comorbidities, such as hypoperfusion, hypertension and diabetes) is a driver of disease pathogenesis. The initial vascular damage is suggested to enhance amyloid accumulation in the brain. Landmark observations in favor of this hypothesis include the emergence of biomarkers related to vascular impairment, prior to the onset of traditional AD biomarkers of amyloid deposition, tau phosphorylation, altered metabolism, structural damage to the brain, and functional impairments to cognition [11]. Recently, induced pluripotent stem cells (iPSCs) harbouring PSEN-1 and PSEN-2 gene variants which were differentiated into brain endothelial-like cells (BECs) demonstrated impairments to bioenergetics, lysosomal acidification, autophagy and oxidative stress in vitro [12]. Extending on this work, we currently lack an understanding of how these putative dysfunctions in BECs from AD patients may be altered by shear stress. The presence of physiological shear stress in modelling the blood-brain barrier (BBB) is significant as it has been demonstrated to not only play a vital role in maintaining healthy BBB phenotype but also lead to vascular dysfunction under aberrant shear conditions [13–15].

In this work, we investigated the impact of static and physiological shear stress conditions on BECs derived from patients harbouring the PSEN-2 N141I gene variant for familial AD (fAD) using a microfluidic platform to model the BBB. Microfluidic approaches to disease modelling are advantageous for their ability to mimic mechanical stimuli as well as to enhance throughput while limiting the use of reagents and cells. Microfluidic approaches are particularly attractive for use in tandem with differentiated iPSCs, where differentiation protocols can take several weeks resulting in limited cell source availability. We report the application and relevance of this microfluidic BBB model system to investigate properties of AD-BECs including barrier permeability, efflux and endocytic transport function, and amyloid toxicity.

## Materials and methods

### Cell culture

H9 human embryonic stem cells (ESCs) were maintained on Matrigel (Corning)-coated 6 well plates in mTeSR1 (STEMCELL Technologies). Human iPSCs were obtained from the New York Stem Cell Foundation repository. 949 (fControl) iPSC line was obtained from a 33-year old female donor with wildtype PSEN-2 genotype and the 950 (AD2) line was obtained from a relative of the fControl line, a 37-year old female donor heterozygous for the N141I PSEN-2 mutation and APOE ε4 mutation (Table S1). iPSCs were expanded and maintained on Geltrex-coated (Thermofisher Scientific) 6-well plates in StemFlex media (Thermofisher Scientific) with daily media changes.

Immortalized human brain microvascular endothelial cells (HBMEC) were maintained in T-75 flasks in EGM-2 media (Lonza) [16]. Media was changed every second day and cells were passaged and collected using trypsin-EDTA. THP-1 monocytes were maintained in T-75 flasks through suspension culture in RPMI-1460 media (Thermofisher Scientific) with 100 IU/mL penicillin-streptomycin and 10% FBS (Gibco). Media was changed every second day.

## iPSC and hESC culture and differentiation

Differentiation of ESCs and iPSCs to BECs have been previously described [17–19]. Briefly, before differentiation, ESCs or iPSCs were singularized with Accutase and plated on Matrigel-coated plates at a density of 4 ~ 8 × 10^4^/cm^2^ in StemFlex supplemented with 10 µM of Y-27,632 (Ray Biotech). At day 0, media was switched to DeSR1 containing DMEM/Ham’s F12 (Thermofisher Scientific), 1 x MEM-NEAA (Thermofisher Scientific), 0.5 x GlutaMAX (Thermofisher Scientific), 0.1 mM β-mercaptoethanol (Sigma), and 6 µM CHIR99021. After 24 h, media was changed to DeSR2 (DeSR1 without CHIR99021) from day 2 to 6. On day 6, media was changed to hESCR1 containing human endothelial serum free media, 20 ng/mL bFGF, 10 µM retinoic acid, and 1X B27. On day 8, cells were dissociated with Accutase and plated at 6 × 10^4^ cells/cm^2^ on 6-well plates coated with human plasma fibronectin (1 mL; 100 µg/mL). On day 10, media was changed to hESCR2 (hESCR1 without bFGF and retinoic acid) for long-term maintenance and daily media changes. After day 10, cells were seeded at confluence for downstream assays at 6 × 10^4^ cells/cm^2^ (Transwell and well plate experiments) or 3 × 10^5^ (VitroFlo experiments). Demonstration of confluency is shown in Fig S2. It is important to note that since the publication of the BEC differentiation protocol [17, 19], which has been widely reproduced by many research groups, new sequencing data has suggested these cells exhibit both epithelial and endothelial features [20, 21]. To ensure the validity of results and relevance to a genuine BEC line, we also compared a subset of these assays to the immortalized HBMEC line which generally showed consistency with fControl-BECs in their response to efflux transport, monocyte adhesion, and endocytic transport. These results are summarized in Fig. S5.

## Fabrication and operation of the microfluidic well plate platform (VitroFlo)

Fabrication of the VitroFlo microfluidic plate has been previously described [22]. Briefly, the plate was fabricated from an injection molded polystyrene well plate top containing reservoir architecture along with a backflow channel geometry to facilitate the recirculation of media. Laser cut double-sized tape (140 μm thick; AR90106 NB, Adhesives Research Inc.) was adhered to the top and bottom layers (CLS450, Universal Laser Systems, Inc) and then used sandwich a porous polyester membrane (PET) with a density of 2.0 × 10^6^ pores/cm^2^ and 1 μm pore size. The double-sided tape layers formed upper and lower channels separated by the porous membrane, on which cells were seeded. A cyclic olefin copolymer (COC) base layer which was laser cut to size was placed underneath to limit leakage and evaporation of media. Channels of the plate were soaked in ethanol for 15 min followed by three washes with PBS followed by UV sterilization for 1 h on each side prior to use. For culture of primary and iPSC-derived BECs, channels were coated with a human plasma fibronectin (30 µL; 100 µg/mL) for 1 h at 37 °C. BECs were then seeded on microfluidic channels at a density of 3 × 10^5^ cells/cm^2^. For permeability, monocyte adhesion, efflux transport function assays, shear stress was applied for 72 h at 12 dynes/cm^2^. Results from these assays were compared against cells cultured under static conditions in Transwell inserts, and while these inserts consisted of the same PET membrane material at the same pore size, we note the difference in scale and media volumes between the two systems may be a potential limitation in direct comparisons between the two platforms.

## Flow cytometry

Cells were harvested using Accutase and fixed using 100% ice-cold methanol for 10 min. Cells were blocked using 40% goat serum in PBS with or without 0.1% Triton X-100 for 20 min at room temperature. Primary antibodies and their dilutions are listed in Table S2. Primary antibodies were prepared in 40% goat serum in PBS and incubated with cells overnight at 4 °C. Cells were then washed three times with PBS containing 5% FBS and incubated with appropriate secondary antibodies at a 1:200 dilution for 30 min at 21 °C. Cells were washed three times with PBS and analyzed on a BD Fortessa flow cytometer. Isotype controls and heat-killed viability controls were used to gate populations. Identification of BEC population was determined through double positive staining of GLUT1 and PECAM1 markers.

## Tranendothelial electrical resistance (TEER) assays

Prior to cell seeding, 6-well Transwell inserts with a 1 μm PET membrane were treated with human plasma fibronectin (1 mL; 100 µg/mL) for 1 h at 37 °C. Cells were seeded on inserts at a density of 6 × 10^4^ cells/cm^2^. After the initial time point at 6 h, barrier measurements were taken at 24-hour intervals for 10 days. To collect measurements, chopstick electrodes were disinfected with 70% ethanol, then soaked in warm media. Electrodes were then placed on either side of the membrane containing the cells and stable readings were collected on a voltohmmeter. TEER values were calculated as TEER = (R_cell_ – R_cell−free_) × surface area of insert. A minimum of 6 measurements per technical replicate were taken across three wells and the experiment was conducted using three biological replicates (here defined as different batches of differentiated cells).

### Immunocytochemistry

Cells were fixed with either 4% paraformaldehyde for 15 min or 100% ice cold methanol for 10 min and washed three times with PBS. Cells were then blocked with 3% BSA for 30 min at 37 °C. Primary antibodies and their dilutions are listed in Table S2. Unless otherwise specified, primary antibodies were incubated at 4 °C overnight followed by secondary antibody incubation at 21 °C for 1 h with agitation. Nuclear counterstain, Hoecht 33342, was used at a concentration of 1 µg/mL for 5 min. Antifade mounting media (Vectashield) was added to fill the volume of the channel for short-term storage. Images were taken on the Olympus FV3000 confocal microscope.

## Western blot

Cells were lysed using Pierce RIPA lysis buffer with 1× Halt protease and phosphatase inhibitors (Thermo Fisher Scientific). Cell lysates were separated using 8–16% Mini-PROTEAN TGX precast protein gels (Biorad) and transferred to nitrocellulose membranes. Membranes were stained with primary antibodies and appropriate loading controls using the staining conditions indicated in Table S2. Antibodies were detected by species-specific, horseradish peroxidase–conjugated secondary antibodies (1:2000) and visualized using a metal DAB substrate reaction kit (Thermofisher Scientific). Colorimetric detection was imaged by the Biorad Chemidoc system. Densitometry analysis was conducted by quantifying band intensity using Image J and fold change of protein abundance of AD-BECs compared to fControl-BECs and the full results are reported in Fig. S4.

## Permeability assay

Permeability was assessed by adding 4 kDa FITC-dextran to the top channel at a concentration of 1 µM. After 1 h at 37 °C, the bottom (recipient) channel or compartment was sampled and fluorescence intensity was measured on a plate reader (Envision 2104 Multilabel Plate Reader, Perkin Elmer). A standard curve was generated by serial dilution of the 4 kDa FITC-dextran stock. The permeability coefficient (P_app_) was calculated using the following equation:

$$\:{P}_{app}=\frac{{\varDelta\:C}_{b}{V}_{b}}{{C}_{i}{A}_{m}\varDelta\:t}$$

where ΔC_b_ is the change in concentration in the recipient channel, V_b_ is the volume of the bottom channel, C_i_ is the initial concentration in the donor channel, A_m_ is the area of the membrane separating the channels, and Δt is the time interval between addition of the tracer and sampling of the recipient channel.

### Monocyte adhesion assays

After exposure to static or shear conditions (12 dynes/cm^2^) for 72 h, BECs were treated with Hoechst 33342-labelled THP-1 monocytes at a concentration of 6.25 × 10^4^ cells/cm^2^ for 1 h. After three washes with PBS, the cells were fixed with 100% ice-cold methanol and stained for GLUT-1 antibody. Some wells were pre-treated with 25 µg/mL of an anti-ICAM-1 antibody (R&D Systems) prior to addition of THP-1 monocytes to block binding to ICAM-1. Three biological replicates with two technical replicates each were performed per sample and ten images were taken per replicate for a total of 60 images analyzed. Monocytes were manually counted on ImageJ, and adhesion is reported as cells/mm^2^.

### Efflux transport assays

P-gp, BCRP, and MRP1 function was evaluated using transporter inhibition studies assessing uptake of fluorescent substrates. Cells were washed once with PBS before pre-treatment with or without each respective inhibitor for 1 h at 37 °C. Cyclosporine A (10 µM, Sigma-Aldrich), KO143 (10 µM, Sigma-Aldrich) and MK571 (1 µM, Sigma-Aldrich) were used for transporter inhibition of P-gp, BCRP, and MRP1, respectively. Cells were then washed with PBS three times and then treated with the respective substrate for 1 h at 37 °C. Rhodamine 123 (10 µM, Sigma-Aldrich), Hoechst 33342 (10 µM, Thermofisher Scientific) and DCFDA (10 µM, Thermofisher Scientific) were used as substrates for P-gp, BCRP, and MRP1, respectively. Each well or channel was washed three times with PBS before cells were collected using Accutase. Cell counts were collected on a cell counter and protein measurements were quantified using the Pierce BCA protein assay (Thermofisher Scientific). Cells were lysed using radioimmunoprecipitation assay buffer (Pierce Biotechnology) containing 1X Halt protease inhibitor cocktail (Thermofisher Scientific). Fluorescence intensity in cell lysates was measured and normalized to total number of cells collected.

### Receptor mediated transport assay (Ac-LDL uptake)

Passage of large molecules across the BBB is regulated through receptor-mediated transport (RMT). To assess endocytic function of BECs, uptake of low-density lipoprotein (LDL) was measured. Cells were treated with 10 µg/mL acetylated-LDL (Ac-LDL) conjugated to BODIPY (Invitrogen) for 4 h at 37 °C. Cells were then washed three times with PBS and fixed with 100% ice-cold methanol. For low density lipoprotein receptor (LDLR) blocking assays, cells were treated with or without a LDLR blocking antibody (R&D systems) at a concentration of 25 µg/mL for 1 h at 37 °C. Cells were stained for LDLR using conditions outlined in Table S2.

### Cell alignment assays

Cells were immunostained for GLUT1 marker using conditions outlined in Table S2 to visualize cell borders. A total of 5 images per channel at 20 X magnification were acquired by confocal microscopy. Cell borders were detected using Trainable Waikato Environment for Knowledge Analysis (WEKA) segmentation plugin on Image-J software (National Institutes of Health, Bethesda, MD) as previously described [22]. In cases where images were unable to result in high quality segmentation, images were validated by manually drawing cell outlines. Aspect ratio along the horizontal axis (direction of flow) as well as frequency distribution of cell alignment angles are reported.

### Aβ oligomer Preparation and cytotoxicity assays

Preparation of Aβ oligomers has been previously reported [23]. Briefly, lyophilized Aβ42 monomers (Cayman Chemical) were resuspended in 1,1,1,3,3,3-Hexafluoroisopropanol (HFIP, Sigma) and lyophilized under sterile conditions. Next, the lyophilized Aβ was resuspended in DMSO to a concentration of 5 mM and sonicated for 10 min. Aliquots were stored at -20 °C until use within two weeks. Twelve hours prior to use, aliquots were mixed with PBS and stored at 4 °C to allow oligomerization to proceed. Aliquots were then diluted with the appropriate cell culture media to achieve the desired final concentration. Cytotoxicity assay kits including MTT, lactate dehydrogenase (LDH) release, and DCFDA cellular ROS were obtained from Abcam and followed as the protocols directed.

### Statistical analysis

Statistical analysis was performed using GraphPad Prism Software. Unless otherwise stated, all studies were performed using a minimum of three independent differentiation batches (biological replicates) with two or three technical replicates. Efflux transport, LDL uptake, cytotoxicity, and monocyte adhesion assays were analyzed using a two-way ANOVA with Sidak post-hoc analysis for multiple comparisons. Western blot densitometry analysis was conducted using nested t-tests. Flow cytometry analysis was conducted using FlowJo (v10.8.1).

## Results

### Validation of BEC differentiation

**Fig. 1:**
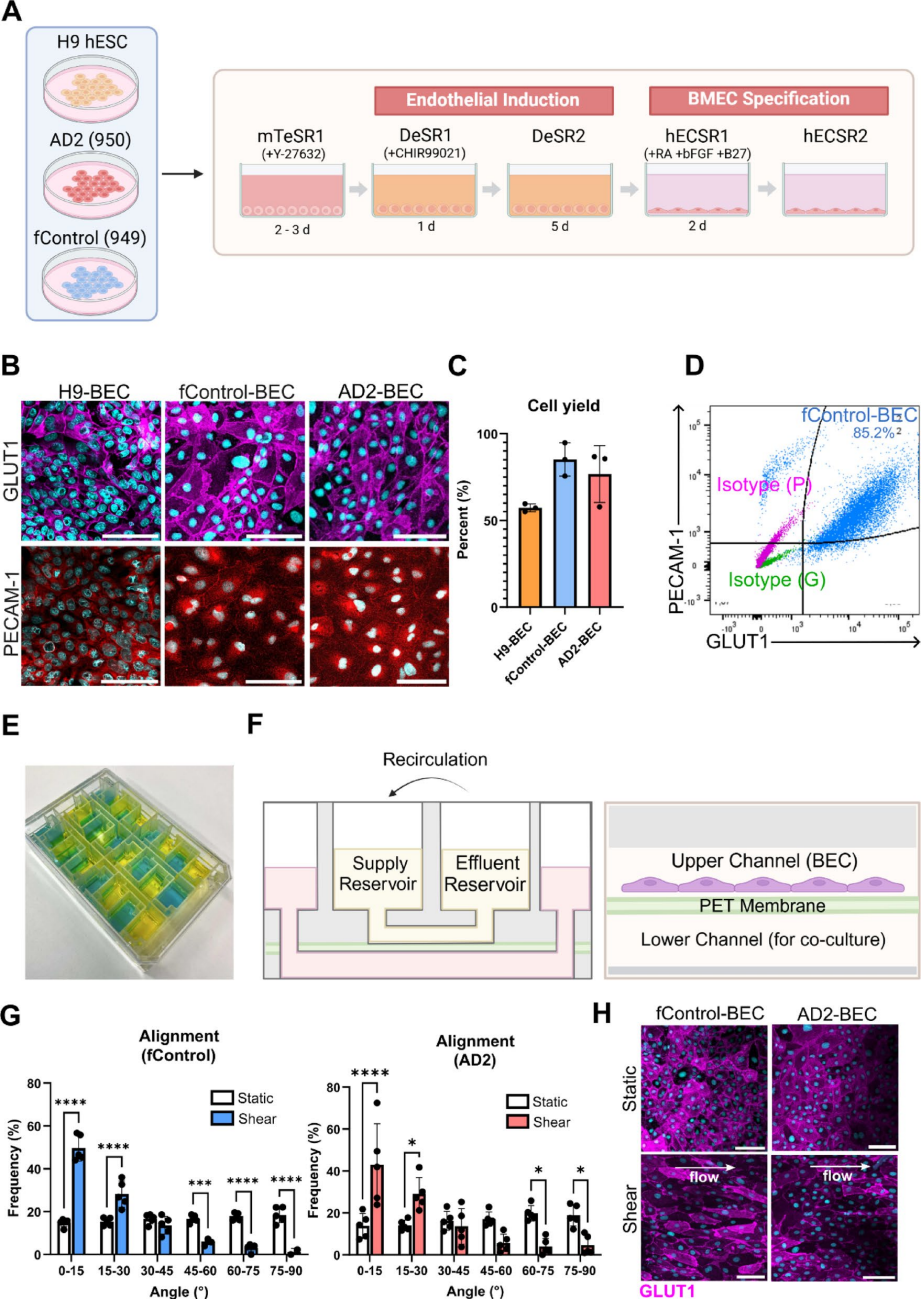
Development of and characterization of H9, AD2 and fControl-BECs in VitroFlo platform. **a** Schematic of BEC differentiation protocol. **b** BECs were characterized by immunocytochemistry assessing for GLUT1 and PECAM-1, scale bar = 50 μm. **c** Cell yield was determined by double positive staining for PECAM-1 and GLUT1 using flow cytometry in three independent differentiation batches. **d** Representative flow plot of fControl-BECs and respective isotype controls for GLUT1 (green) and PECAM-1 (purple) is shown.**e** Isometric view of the VitroFlo microfluidic well plate depicting the different modules filled with coloured dyes.**f** Schematic diagram of a single module of the VitroFlo microfluidic well plate. Cell alignment studies were conducted by growing BECs under shear or static conditions.**g** BEC alignment in the direction of shear flow was quantified using Image J and ** h ** Representative immunocytochemistry images used for cell alignment studies are shown with GLUT1 staining used to identify cell borders (magenta). White arrow indicates direction of flow. Scale bar = 100 μm

A schematic diagram of the differentiation protocol used is shown in Fig.1A. The H9 hESC line was used as a benchmark control, as it has been previously published under the BEC differentiation scheme [17,18]. Flow cytometry was used to validate the percentage yield of the cells through double positive staining of GLUT1 and PECAM-1 markers. The flow cytometry gating strategy is shown in Fig. S1 and a representative flow cytometry plot is shown in Fig. 1B. A yield of 58.2 ± 2.3% was achieved for H9-BECs, similar to that of previously reported values [18], whereas the iPSCs showed much higher yields of 85.2 ± 9.6% and 76.8 ± 16.4% for fControl-BECs and AD2-BECs, respectively (Fig. 1C). GLUT1 and PECAM-1 markers were also confirmed visually using immunocytochemistry (Fig. 1D)

### Implementation of cells on the VitroFlo

To evaluate the suitability of the VitroFlo platform in inducing shear-dependent changes to iPSC-BECs, cell alignment studies were conducted. fControl and AD2-BECs were seeded at 3 × 10^5^ cells/cm^2^ and cultured under shear or static conditions for 72 h. After shear or static conditioning, cells were fixed and stained for GLUT1 cell border marker. Both cells exhibited an elongated appearance along the direction of flow (Fig. 1H) and a significant increase in alignment (Fig. 1H) and aspect ratio (Fig. S3) with respect to direction of flow. Specifically, there was a significant increase in alignment between 0–15° (fControl: 15% in static vs. 50% in shear, *p* < 0.0001; AD2: 14% in static vs. 43% in shear, *p* < 0.0001) and 15–30° (fControl: 15% in static vs. 28% in shear, *p* < 0.0001; AD2: 14% in static vs. 29% in shear, *p* = 0.0166). These results are consistent with previous reports showing alignment in HUVECs [22], demonstrating reproducibility of morphological shear responsive characteristics in fControl- and AD2-BECs.

### Characterization control and AD barriers

**Fig. 2:**
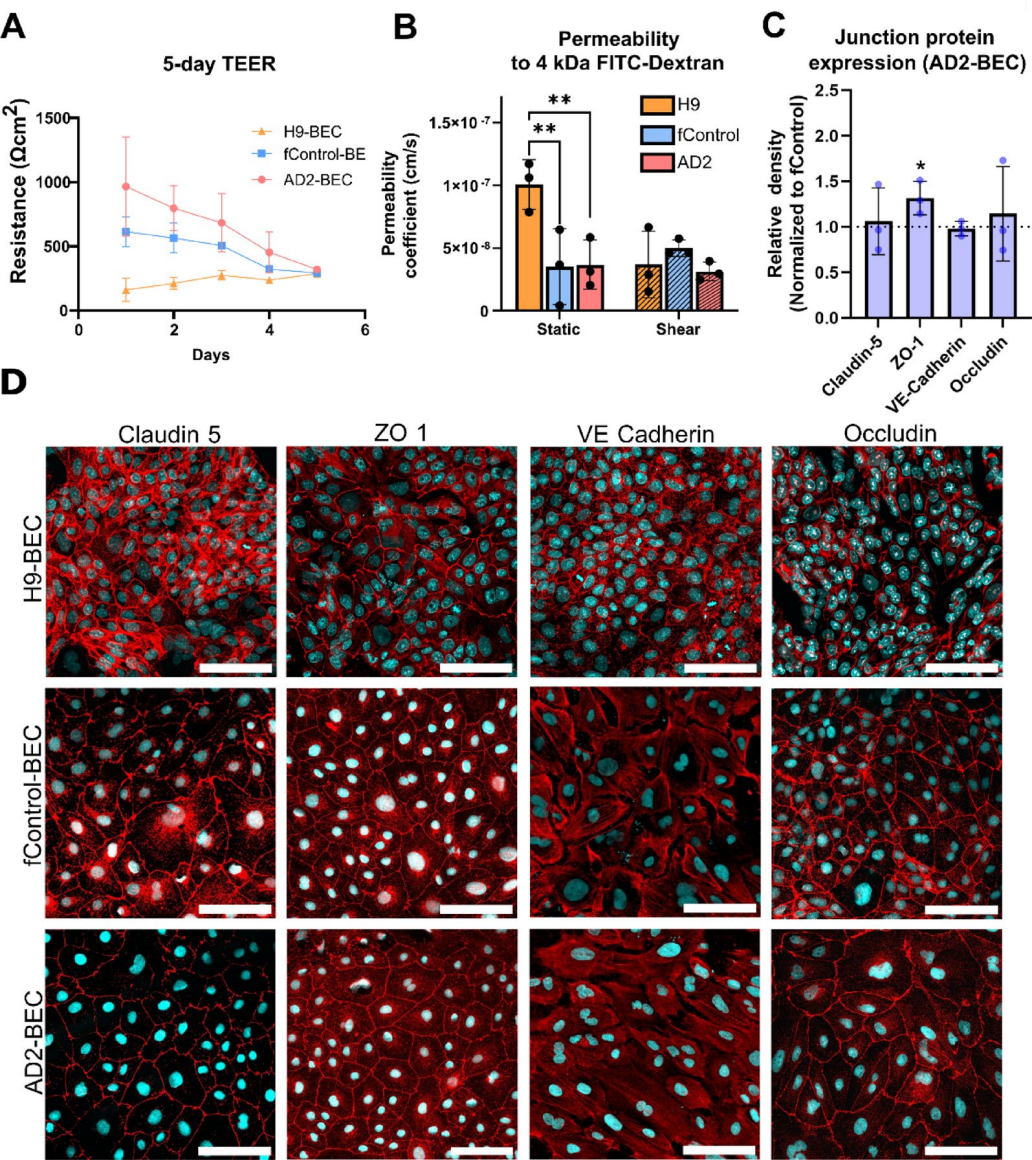
Barrier characteristics of H9,AD2,and f Control BECs. **a** Plots of transendothelial electrical resistance measures over 5 days post-seeding in three independent differentiation batches (*n* = 3) with three technical replicates. **b** Permeability coefficients of 1 µM 4 kDa FITC-dextran in H9, AD2, and fControl BECs in three independent differentiation batches (*n* = 3) with three technical replicates. (H9 - fControl: *p* = 0.006; H9 – AD2: *p* = 0.007). **c** Densitometry analysis of junction proteins detected by western blot. Fold change of protein expression band density in AD2-BECs relative to fControl-BECs is reported in three independent differentiation batches (*n* = 3). **d** Representative immunocytochemistry images of junction proteins (claudin-5, occludin, VE-cadherin and ZO-1) in H9, AD2, and fControl BECs, scale bar = 100 μm

To assess the integrity of the barriers formed by AD2 and fControl-BECs, measurements of TEER values and permeability to 4 kDa FITC-dextran were performed (Fig. 2A). As the sizes of the inlet and outlet channels of the VitroFlo plate limited the ability for the TEER electrodes to take accurate measurements, TEER was performed under static conditions in Transwell inserts (1 μm pore size). Both fControl-BECs and AD2-BECs achieved their maximum TEER values 24 h after cell seeding at 614 ± 116 Ωcm^2^ and 966 ± 387 Ωcm^2^, respectively. However, H9-BECs did not achieve their maximum TEER value until 5 days post seeding at 286 ± 19 Ωcm^2^. By day 5, there were no significant differences in TEER values across all three cell lines. To assess the paracellular permeability of the barriers, fluorescent tracer assays were performed. Briefly, 24-well Transwell inserts or the Vitroflo platform was seeded with BECs and exposed to static or shear conditions, respectively, for 72 h. After static or shear conditioning, 1 µM of 4 kDa FITC-dextran was added to the top channel as a fluorescent tracer. After 1 h, the bottom channel was sampled, and fluorescence was measured in a plate reader. Under static conditions, H9-BECs show significantly higher permeability compared to fControl and AD2-BECs (*p* = 0.006, *p* = 0.0072, respectively), but there were no significant differences between fControl-BECs and AD2-BECs. Under shear stress conditions, there were no significant differences across all three cell lines (Fig. 2B). Lastly, to evaluate junction proteins involved in maintaining the integrity of the barrier, western blot and immunocytochemistry was performed for markers claudin-5, occludin, ZO-1 and VE-cadherin. Densitometry analysis of western blots demonstrated no significant differences in junction protein expression between fControl-BECs and AD2-BECs grown in static conditions (Fig. 2C). Claudin-5, occludin, and ZO-1, and VE-cadherin, were expressed at cell-cell junctions in all three cell lines (Fig. 2D). Taken together, these results suggest that barriers formed between fControl and AD2-BECs are similar in terms of their barrier integrity, permeability, and junction protein expression. Interestingly, unlike the H9-BECs, permeabilities of both the fControl and AD2-BECs were not sensitive to shear stress pre-conditioning.

### Assessment of monocyte adhesion

**Fig. 3:**
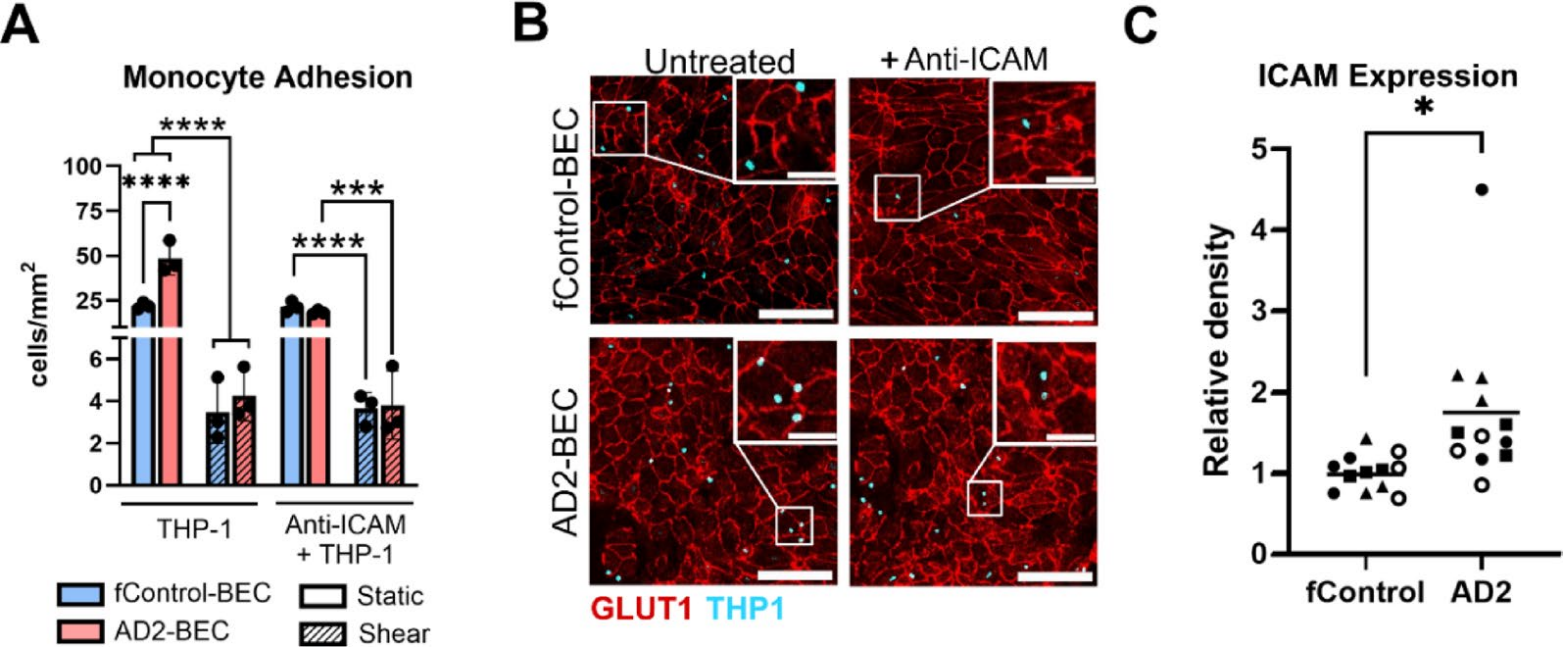
THP-1 monocyte adhesion on AD2 and f Control-BEC monolayers. BEC monolayers were left untreated or treated with 25 µg/mL of anti-ICAM-1 antibody for 1 h. **a** Counts obtained from manually counting Hoechst-labelled THP-1 monocytes after 1 h of attachment onto EC monolayers were reported as cells/mm^2^. A total of three independent differentiation batches and two technical replicates were used with ten images analyzed from each sample group (fControl - AD2: *p* < 0.0001; static-shear *p* < 0.0001, *p* < 0.001). **b** Representative images of THP-1 monocyte adhesion onto EC monolayers, scale bar = 150 μm (50 μm for inset). Endothelial borders were stained using an anti-GLUT-1 antibody (in red). **c** Densitometry analysis of ICAM-1 expression in four biological replicates of fControl and AD2-BECs shows a significant enhancement of ICAM-1 in the AD2 line (*p* = 0.0392)

The endothelium of the central nervous system (CNS) tightly regulates the trafficking of inflammatory cells in physiological conditions. As such, we looked to explore the degree of leukocyte adhesion onto the barriers generated by fControl-BECs and AD2-BECs. Using Hoechest-labelled THP-1 monocytes, monocyte counts after 1 h of incubation were manually performed on BEC monolayers and reported as cells/mm^2^. An anti-ICAM-1 antibody was used as a negative control to block interactions with the THP-1 cells. Under static conditions, monocyte adhesion was significantly enhanced on AD2-BEC barriers as compared to fControl-BEC barriers (*p* < 0.0001; Fig. 3A–B). The enhanced adhesion corresponded with increased ICAM-1 expression in AD2-BECs, as determined by western blot (*p* = 0.0392; Fig. 3C) and immunostaining (Fig. S6). However, under shear conditions, a reduction in monocyte adhesion was observed in both lines after 72 h of shear conditioning (fControl-BEC static-shear: *p* < 0.0001; AD2-BEC static-shear: *p* < 0.0001; Fig. 3A), with no significant difference in adhesion observed between fControl-BECs and AD2-BECs (*p* = 0.9981; Fig. 3A).

### Assessment of BEC transporter function

**Fig. 4:**
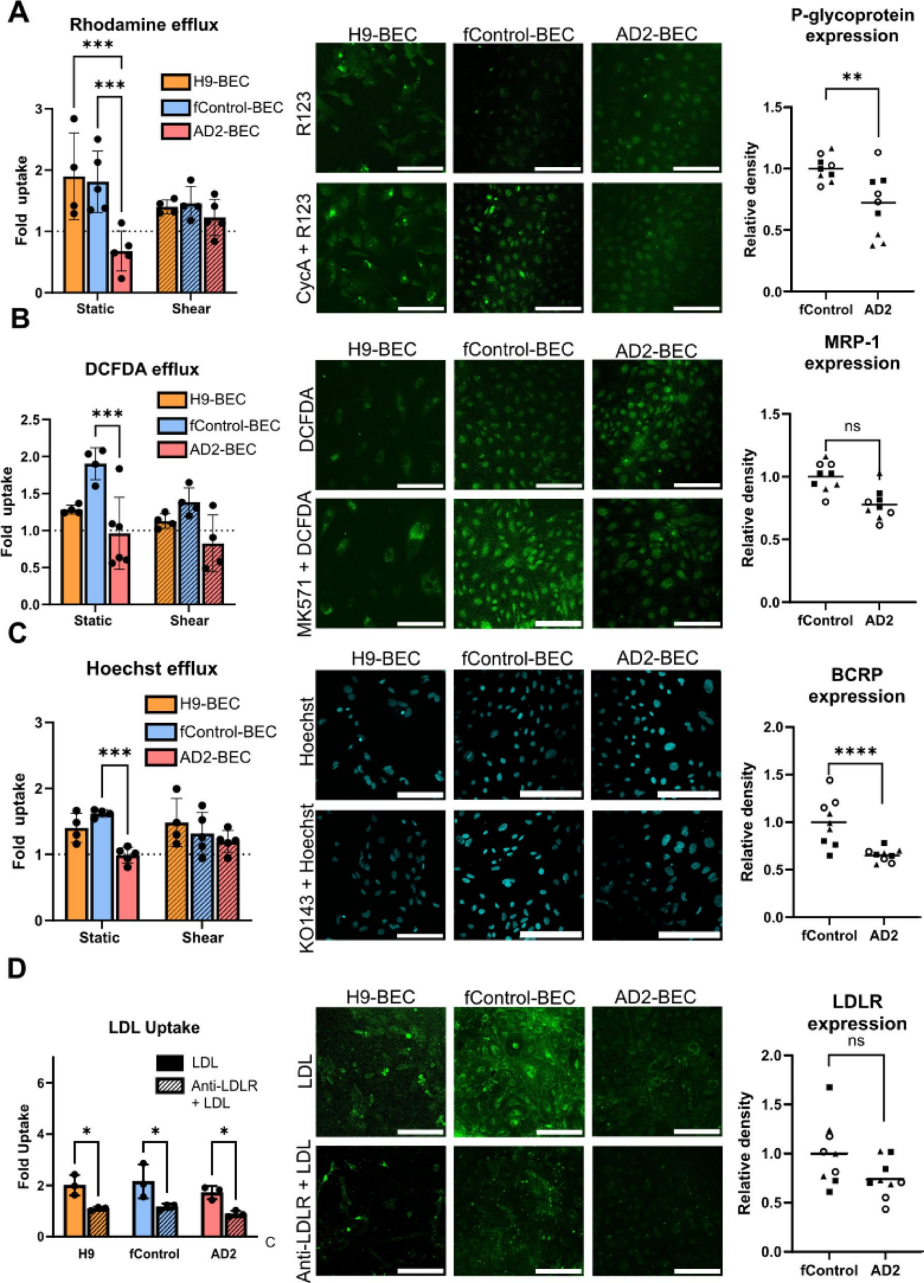
Transport function of H9, f Control, and AD2-BECs. Efflux transport function was assessed using transporter inhibition assays. All results are reported as fold change in substrate uptake relative to non-inhibited controls, treated with the substrate. **a** Assessment of p-glycoprotein (P-gp) function was performed using rhodamine 123 uptake and inhibiting P-gp with 10 µM of cyclosporine A where AD2-BECs exhibit reduced P-gp function compared to controls (Static H9 – Static AD2: *p* = 0.0012; Static fControl – Static AD2: *p* = 0.0016) and reduced transporter expression (*p* = 0.0203). **b** Assessment of MRP1 function was performed using 10 µM of DCFDA and inhibition with MK571 which revealed reduced MRP1 function in AD2-BECs compared to controls (Static fControl – Static AD2: *p* = 0.0004) but with no significant difference in expression levels (Static fControl – Static AD2: *p* = 0.2583). **c** Assessment of BCRP function was performed using 10 µM Hoechst and inhibition with KO143 which revealed reduced BCRP function by AD2-BECs compared to controls (Static fControl – Static AD2: *p* < 0.0001) and reduced transporter expression (Static fControl – Static AD2: *p* = 0.0372). **d** LDL uptake was evaluated in cells using 10 µg/mL of Ac-LDL BODIPY after 4 h. Endocytosis of LDL was blocked using an anti-LDLR antibody. Results are reported as fold change in uptake compared to untreated controls (H9: *p* = 0.0193; fControl: *p* = 0.0054; AD2: *p* = 0.0179). Representative images were uniformly adjusted within each cell line and substrate group for brightness and contrast to better visualize fluorescent signal. All scale bars = 150 μm

The BBB exhibits a unique system of carrier-mediated transporters to tightly regulate the exchange of important biomolecules such as amino acids, glucose, nucleosides, peptides, and organic molecules while limiting the entry of potentially harmful xenobiotics. Here, we investigated the function of three ATP-binding cassette (ABC) transporters (P-gp, BCRP, and MRP1) to evaluate their efflux transporter activity, as well as activity of low-density lipoprotein receptor (LDLR) to evaluate clathrin-mediated endocytosis function. Briefly, BEC monolayers were treated with or without respective small molecule inhibitors, prior to treatment with substrates for each transporter (Rhodamine 123 for P-gp, Hoechst 33342 for BCRP, and DCFDA for MRP1). Results are reported as fold change in uptake of each substrate relative to the un-inhibited control (Fig. 4A-C). Intriguingly, AD2-BECs show a significant reduction in uptake of all three substrates when the receptor was inhibited when compared to fControl-BECs (Rhodamine: *p* = 0.0017, Hoechst: *p* = 0.0002, DCFDA: *p* = 0.0004). These results suggest impairment of efflux transport in AD cells and were further supported by an observation of decreased P-gp and BCRP expression in AD2-BECs as compared to fControl (P-gp: *p* = 0.0203, BCRP: *p* = 0.0372), but not for MRP1 (*p* = 0.2583). Interestingly, after shear conditioning, the difference observed in substrate uptake by AD2-BECs was no longer apparent, suggesting shear stress may play a protective role in maintaining efflux transport function that is lost when the cells lack exposure to physiological flow.

To evaluate clathrin-mediated endocytosis, 10 µg/mL of Ac-LDL tagged with BODIPY was incubated with the cells for 4 h after treatment with or without an anti-LDLR antibody to block LDLR activity. All three cell lines showed functional endocytosis that was dependent on interactions with LDLR, as demonstrated through a reduction in uptake in blocked conditions (H9: *p* = 0.0193; fControl: *p* = 0.0054; AD2: *p* = 0.0179; Fig. 4D). As no significant differences were observed statically conditioned cells from disease and control groups, Ac-LDL uptake studies were not performed in the shear conditions. Finally, there were no significant differences between fControl and AD2-BECs in LDLR expression from cells grown in static conditions.

#### Toxicity of BECs to amyloid oligomers

**Fig. 5:**
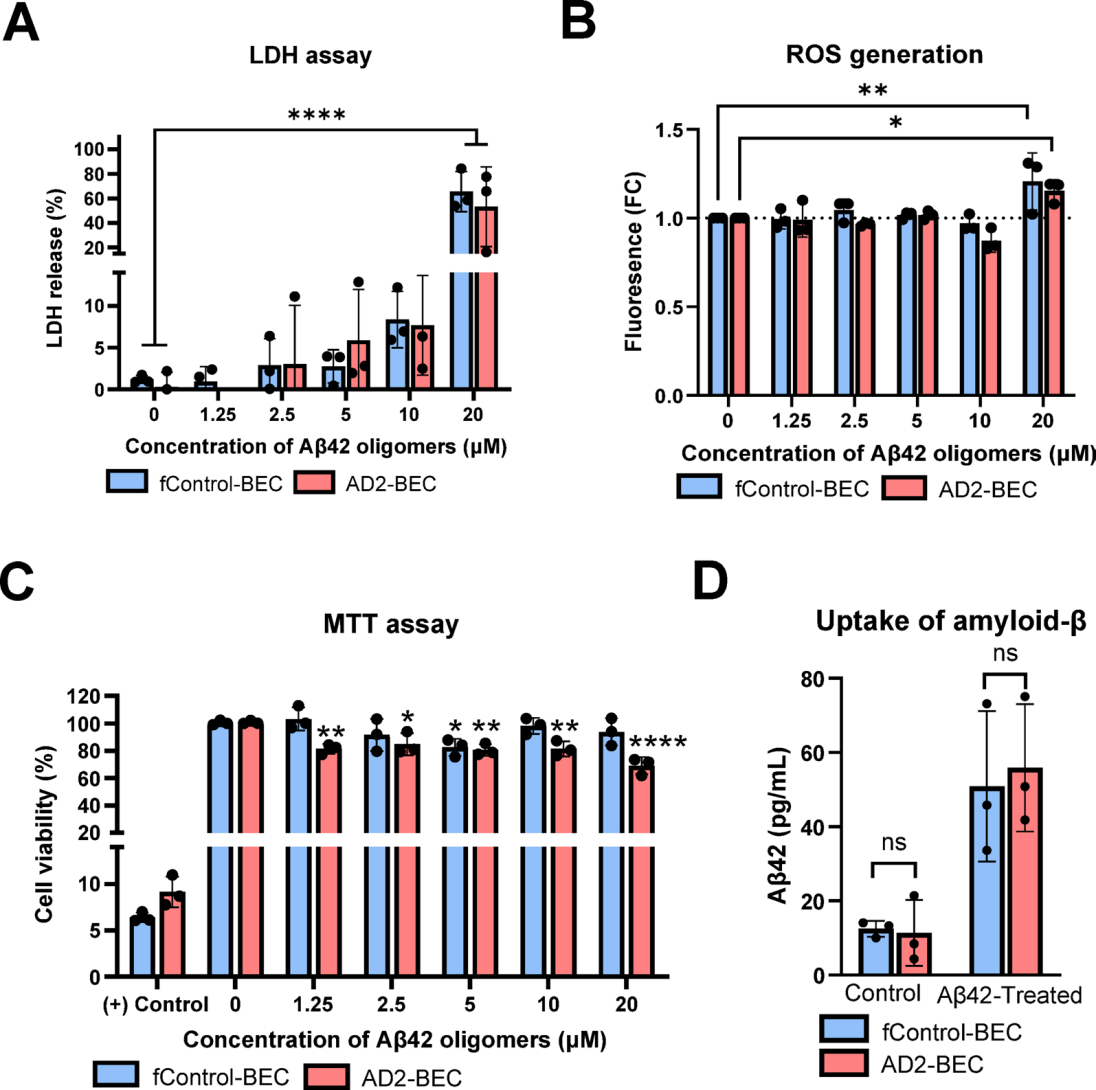
Cytotoxicity response of fControl and AD2-BECs to Aβ42 oligomers and monomers. A concentration range of 0 ~ 20 µM A$\:\beta\:$ oligomers was prepared and treated on EC monolayers for 72 h. **a** LDH, **b** ROS, and **c** MTT assays were performed to assess cell viability. 20 µM of A$\:\beta\:$ oligomers demonstrated to induce significant LDH release (*p* < 0.0001) and ROS generation (fControl: *p* = 0.0044; AD2: *p* = 0.0477). There was a significant reduction in metabolic activity by AD2-BECs, as demonstrated by reduced viability in AD2-BECs, however, this did not appear to be driven by enhanced ROS production.** d** After 72 h of exposure, both lines did not show any significant differences in A$\:\beta\:$ oligomer uptake as measured by ELISA

As amyloid has been widely studied as a major player in AD pathogenesis, we explored the toxicity of Aβ42 oligomers in fControl and AD2-BEC monolayers in static conditions. A concentration range of 0–20 µM of oligomers was prepared and used to treat cells for 72 h. Using an LDH assay as a measure of cellular membrane integrity, both cell lines showed significantly increased LDH release at 20 µM (*p* < 0.0001; Fig. 5A). To determine if amyloid induced ROS generation, a DCFDA assay was performed and demonstrated, similar to the LDH assay, ROS generation was significantly increased in both lines at 20 µM (*p* < 0.0001; Fig. 5B). Lastly, an MTT assay was performed as a measure of cell metabolic activity, where interestingly, AD2-BECs exhibited reduced viability across all concentrations of oligomers tested, compared to the untreated controls (Fig. 5C). To determine if this altered response was due to differences in oligomer uptake by the cells, the cells were lysed and an ELISA was performed, however, no significant differences were observed, both in baseline amyloid production as well as amyloid uptake after treatment. (Fig. 5D). Taken together, this suggests that AD2-BECs have increased sensitivity to Aβ oligomers, however, the precise mechanisms driving these differences remain to be explored.

## D**iscussion**

Several therapeutic candidates that have shown promise against AD in animal models have failed in human patient trials [23]. In vitro models offer advantages of lower cost and higher throughput, however, have traditionally faced limitations in terms of biological relevance. The advancement of two- and three-dimensional, multicellular, dynamic models has given headway for generating in vitro systems that capture complex disease hallmarks and relevant microenvironmental cues. There is an ongoing need to develop these tools in pursuit of better predictive validity for drug screening. To this end here we generated microfluidic models using patient stem cells which demonstrate alterations to immune cell attachment, efflux transporter function, and cytotoxicity to Aβ42 oligomers in BECs derived from the AD2 PSEN-2 N141I iPSC line.

Microfluidic platforms allow for the application of shear stress forces to mimic mechanical stimuli of blood flow in the brain. Shear stress is critical to regulating and maintaining BEC phenotype by upregulating transporter and cell-cell junction gene expression [13, 24] reducing leukocyte adhesion molecule expression [25], altering bioenergetic pathways to favor more efficient aerobic respiration [13], enhancing barrier strength [26–28], reducing permeability [13, 29], and altering nitric oxide production [25]. Different levels of shear stress have also been shown to confer different BBB phenotypes. For example, high pulsatile shear stress (40 dynes/cm^2^) has been shown to deteriorate primary HBMECs by decreasing expression of junction proteins and altering their morphology. In contrast, physiological levels of 10–20 dynes/cm^2^ have been demonstrated to enhance transporter and junction protein expression [14]. Another study observed primary mouse endothelial cells exposed to 1 ~ 25 dynes/cm^2^ over a period of 24–96 h showed higher junction protein expression and TEER than those grown statically or exposed to >25 dynes/cm^2^ shear stress [30]. Application of a shear level of 12 dynes/cm^2^ has been previously demonstrated to reflect physiological conditions in capillaries and has reproduced morphological and functional changes in endothelial cell lines over a short period of time (72 h) and thus was selected for our studies [31, 32].

While barriers generated by fControl and AD2 iPSCs appear to be similar in morphology and integrity through initial evaluation of TEER and permeability, this work has demonstrated AD-associated perturbations to BEC function in areas of drug transport, immune cell trafficking, and cytotoxicity. Further, the observed dysfunction of efflux transport and enhanced monocyte adhesion in static conditions was mitigated after shear conditioning, suggesting that the lack of shear stress results in enhanced immune reactivity of the endothelium. It may be argued that in vitro modelling under static conditions closer mimics a hypoperfusion state in which the lack of shear flow results in the deleterious effects of AD becoming pronounced. Within the context of hypoperfusion, in vivo models have demonstrated hypoperfusion aggravates impairment of the BBB in AD. Mouse models and clinical observations of cerebral hypoperfusion observed neurovascular dysfunction by reduction of P-gp expression [33, 34], reduction of tight junction proteins [35], reduction in pericytes [36], increased permeability [35], alterations to amyloid transport mechanisms [37] and increased Aβ deposition [33, 37, 38]. Future investigation into assessing various shear stress levels and durations may be of importance to better understand this phenomenon.

In order to evaluate interactions of the barriers with immune cells, we evaluated monocyte adhesion and observed enhanced monocyte adhesion and increased ICAM-1 expression by AD2-BECs. Inflammatory processes have been long shown to play a role in AD pathogenesis through activation of complement and alternative pathways, enhanced cytokine and chemokine secretion, and microglial activation [39–41]. More recently, the role of adhesion molecules has been studied in AD for its relevance in amyloid metabolism, microglial activation and processing, and neuronal plasticity [42, 43]. Clinically, ICAM-1 has been found to be localized to blood vessels near neuritic plaques of AD patients [42–44]. Further, ICAM-1 has been demonstrated to have higher immunoreactivity in the cerebral vasculature of AD patients as compared to healthy controls [44]. These findings have also been reported in vitro where primary human BECs exposed to amyloid show time-dependent increase in ICAM-1 expression [45]. These results may also have implications in addressing controversies in ICAM expression in AD models. For example, aged mice models alone do not show alterations in ICAM or VCAM expression [46], which may highlight the importance of AD specific phenotype and mutation to observe clinically relevant changes to adhesion molecule and subsequent impacts to microglial recruitment and amyloid metabolism. Exploration of other leukocyte types and adhesion molecules may provide further insight, in addition to exploring clustering and localization of such molecules in response to shear forces [47].

Next, we sought to evaluate efflux transporter function in AD-BECs. The function of transporters is of particular interest as transporter dysfunction has been observed in patients with AD [48, 49] and new evidence suggests that efflux transporters such as P-gp and BCRP play an important role in the removal of toxic amyloid species [50–52]. In our work, we demonstrated that the PSEN-2 N141I mutation results in impairment to P-gp, BCRP, and MRP1 function, with corresponding decreases in P-gp and BCRP expression. Raut et al. also observed impaired MRP1 function in iPSC-derived BECs harbouring the PSEN-2 N141I mutation, but did not observe impairments to P-gp and BCRP function, perhaps due to differences in patient cell sources [12] While the mechanisms involved in how fAD could induce alterations to transporter expression remains unknown, there is evidence suggesting amyloid itself could be involved in modulating transporter activity and expression. In mice subcutaneously injected with Aβ42, decreases in P-gp expression, but not BCRP expression were observed, suggesting Aβ42 can downregulate transporters [53]. Clinically, it has been suggested that non-demented patients show increased P-gp expression, potentially as a compensatory result to increase Aβ clearance, whereas in patients with later stages of cerebral amyloid angiopathy (CAA), P-gp was found to be reduced, and even lost in some patient vessels [54]. Dissecting the mechanistic basis of transporter dysfunction in fAD will be important to understanding alterations to amyloid trafficking as well as investigating therapeutic effects of modulating efflux transporters to reduce amyloid burden.

Finally, we investigated the cytotoxicity of our model to Aβ oligomers. There is a significant body of evidence to demonstrate AD-derived neuronal cells are more susceptible to the toxic effects of amyloid and these variations in cytotoxicity are gene variant dependent [55–60]. The AD2 line harbouring the PSEN-2 N141I variant has been reproducibly shown to increase Aβ42/40 ratio and alter cell viability in iPSC-derived neuronal cells as well as transfected cell lines [61–66]. Through conducting an MTT viability assay, decreased viability of Aβ on AD2-derived BECs relative to fControl-BECs was observed at all Aβ oligomer concentrations tested (Fig. 5C). Further, assessments using LDH and ROS generation assays demonstrated compromised membrane integrity and enhanced ROS generation in AD2-BECs treated with 20 µM Aβ oligomers (Fig. 5A, B). While the precise mechanisms of cytotoxicity remain to be further explored, induction of inflammatory signaling pathways may offer some clues. Aβ itself has been demonstrated to induce proinflammatory cytokine secretion and acts synergistically with inflammatory factors to promote inflammation [67–69]. Further, this gene variant has been implicated in dysregulation of calcium homeostasis [70–73], inflammation [74, 75], and apoptosis [65]. Mitochondrial dysfunction may be a contributor to cytotoxicity where differential expression of oxidative phosphorylation genes in AD patients was found to be implicated in mitochondrial dysfunction and hypometabolism [76, 77]. Future work on investigating differences in cellular trafficking of amyloid and cell signaling activity may help to better understand cell line dependent differences.

Potential limitations of this work that should be considered include the relevance of iPSC-derived BEC lines. As aforementioned, the differentiation protocol used in this study has been demonstrated to yield cells with both epithelial and endothelial-like properties. While in our work we characterize the endothelial-like properties of these cells, including barrier integrity (TEER, permeability), characteristic BEC marker expression, and BBB functions (efflux transport and monocyte transmigration), we have yet to investigate the epithelial properties of these cells. Additionally, we observed discrepancies in TEER measurement in the H9-BEC group compared to previously reported literature [19, 78], potentially due to variations in seeding density and culture platform, For example, BEC differentiation is sensitive to cell seeding density [19], with maximal TEER reported at 35,000 cells/cm^2^, which is less than the seeding density used in our study. We also noted lower immunostaining intensity of ICAM-1 in the iPSC-derived BECs compared with immortalized HMBECs (Fig. S5). While the cells were also positive for the tested junction markers, we also note a lack of continuous staining of VE-cadherin localized to the cell junctions. Overall, these points highlight the need for improved strategies for directed differentiation of organotypic ECs to promote reproducibility and limit batch to batch variation. Recently, new protocols have been developed refining the generation of iPSC-BECs for improved consistency and relevant marker expression [79–81]. Using defined differentiation protocols and further refining conditions, such as implementing cocultures with astrocytes and other support cells of the NVU, is an important direction of this work. Additionally, this work is currently limited in its focus on the PSEN N141I gene variant. While this gene variant remains relevant due its reported alterations to cells of the NVU [82, 83] and associations with the development of cerebral amyloid angiopathy [84, 85], further exploration of the impact of other gene variants of AD will be of importance to identify whether the findings of exacerbated AD phenotype under a hypoperfused state are observed in other AD models.

## Conclusion

In this study, we derived BECs from a donor with an fAD gene variant and their unaffected family member, and implemented them for study in a microfluidic model of the BBB. We, for the first time, demonstrated functional changes in PSEN-2 N141I iPSC-BECs related to efflux transport, immune cell adhesion, and amyloid toxicity in both static and shear stress conditions. Further, we observed that the lack of shear stress, mimicking a hypoperfusive state, associated with impairments in vascular function by AD2-BECs. In contrast, application of shear stress maintained critical BEC functions suggesting normal shear stress may play a protective role in maintaining BBB function in AD. Taken together, these results highlight the need for models that not only investigate nascent properties of BECs but also provide adequate microenvironmental cues such as fluid shear stress with implications towards unravelling the mechanisms underlying vascular dysfunction in neurodegenerative disease.

## Supplementary Information

Below is the link to the electronic supplementary material.

Supplementary Material 1

## Data Availability

All data generated and analyzed during this study are included in this published article and its supplementary files. Any additional data is available from the corresponding author upon reasonable request.

## Abbreviations

Aβ: Amyloid beta
ABC: ATP-binding cassette
Ac-LDL: Acetylated low density lipoprotein
AD: Alzheimer’s disease.
AD-BEC: Alzheimer’s disease brain endothelial-like cell
APP: Amyloid precursor protein
BBB: Blood-brain barrier
BCA: Bicinchoninic acid
BCRP: Breast cancer resistant protein
BEC: Brain endothelial-like cell
CAA: Cerebral amyloid angiopathy
COC: Cyclic olefin copolymer
DCFDA: 2’7’-dichlorofluorescin diacetate
EC: Endothelial cell
ESC: Embryonic stem cell
fAD: Familial Alzheimer’s disease
HBMEC: Human brain microvascular endothelial cells
HFIP: 1,1,1,3,3,3-hexafluoroisopropanol
HUVEC: Human umbilical vein endothelial cell
ICAM-1: Intracellular adhesion molecule-1
iPSC: Induced pluripotent stem cell
LDH: Lactate dehydrogenase
LDL: Low-density lipoprotein
LDLR: Low-density lipoprotein receptor
MRP1: Multidrug resistance-associated protein 1
MTT: 3-[4,5-dimethylthiazol-2-yl]-2,5 diphenyl tetrazolium bromide
PBS: Phosphate buffered saline
PECAM-1: Platelet and endothelial cell adhesion molecule-1
PET: Polyester
PSEN-1: Presenilin-1
PSEN-2: Presenilin-2
ROS: Reactive oxygen species
TEER: Transendothelial electrical resistance
VCAM: Vascular cell adhesion molecule 1
