# Supplementary material for: Alzheimer’s disease PSEN-2 N141I mutation reveals altered and shear-sensitive brain endothelial cell-like phenotype in human iPSC-derived models
Source: Acta Neuropathol Commun. 2025 Nov 11;13:231. doi: 10.1186/s40478-025-02152-3 (PMC12607174; doi:10.1186/s40478-025-02152-3)
Supplement: Supplementary file 1 — Supplementary Material 1 [file 40478_2025_2152_MOESM1_ESM.pdf]

SUPPORTING INFORMATION

Alzheimer's Disease PSEN-2 N141I mutation reveals altered brain endothelial cell phenotype in human-iPSC derived models

Lily Takeuchi[1,2*], Jennifer Lam[2], Craig A. Simmons[1-3*]

[1]Institute of Biomedical Engineering, University of Toronto, 164 College Street, Toronto, ON, M5S 3G9

[2]Translational Biology and Engineering Program, Ted Rogers Centre for Heart Research, 661 University Ave, Toronto, ON, Canada, M5G 1X8

[3]Department of Mechanical and Industrial Engineering, University of Toronto, 5 King's College Road, Toronto, ON, Canada. M5S 3GB

***Co-corresponding Authors:** c.simmons@utoronto.ca; lily.takeuchi@mail.utoronto.ca

List of figures

Fig. S1 Flow cytometry gating strategy

Fig. S2 Confirmation of BEC monolayer confluency

Fig. S3 Aspect ratio quantification of BECs exposed to static and shear flow conditions

Fig. S4 Representative western blots and corresponding densitometry analysis

Fig. S5 Characterization of immortalized HBMECs for comparison with differentiated brain endothelial-like cells

Fig. S6 Representative images of ICAM-1 immunostaining on iPSC-derived BECs

List of tables

Table S1 iPSC and hESC cell line information

Table S2 Staining conditions of antibodies used for western blot and immunocytochemistry

Table S3. Efflux transport receptor inhibitors and substrates

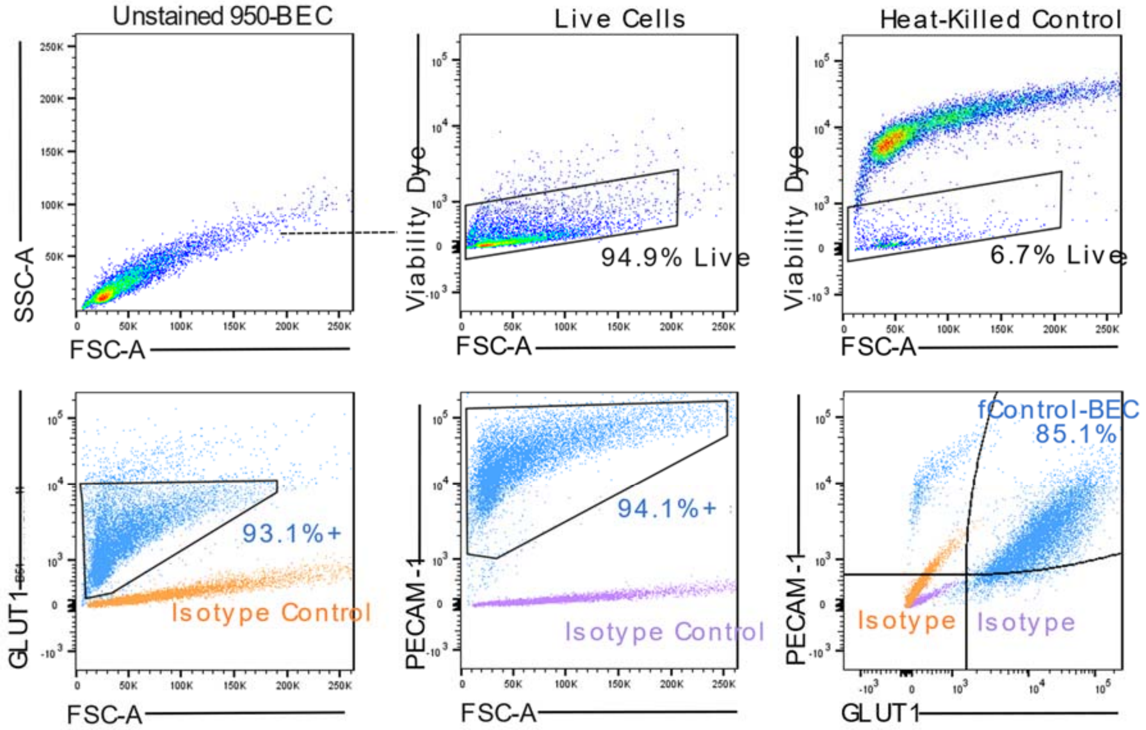

Fig. S1 Flow cytometry gating strategy. A representative flow cytometry gating analysis is shown for fControl-BEC. Double positive staining of GLUT-1 and PECAM-1 markers was used to determine cell yield post-differentiation of BECs. Exclusion of doublets was performed on the SSC-A – SSC-H axis. Next, a fixable viability dye was used to exclude dead cells and gating was determined using a heat-killed control sample. Mouse IgG2a kappa and rabbit IgG isotype controls for GLUT1 and PECAM-1, respectively, were incubated at the same concentrations as the corresponding primary antibody and used for gating.

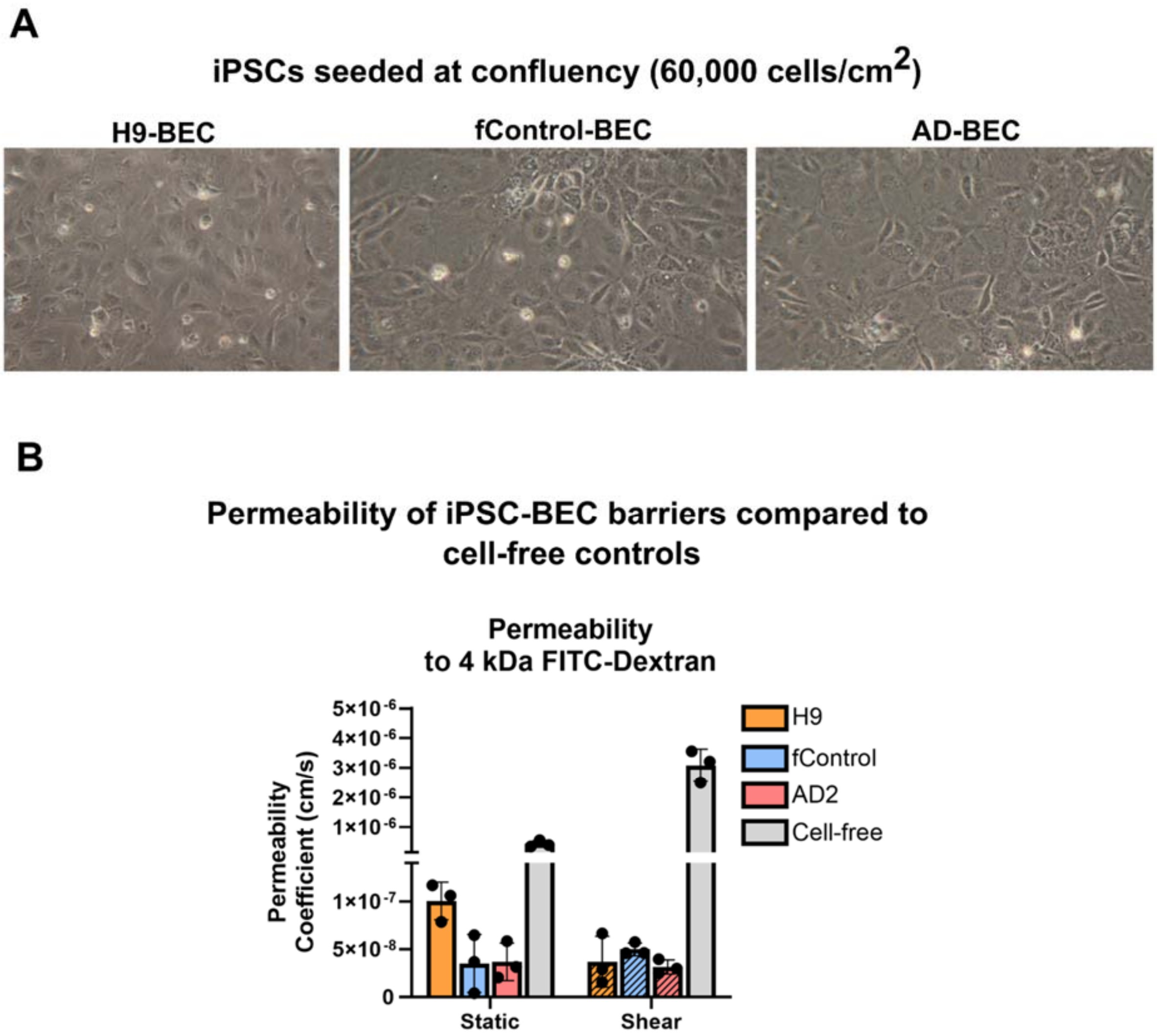

Fig. S2 Confirmation of BEC monolayer confluency. A) Bright field images of BECs post day 10 differentiation to demonstrate confluency at assay seeding density (60,000 cells/cm^2). B) Permeability measurements in comparison to cell-free Transwell inserts (for static condition) and VitroFlo platform (for shear condition) are shown.

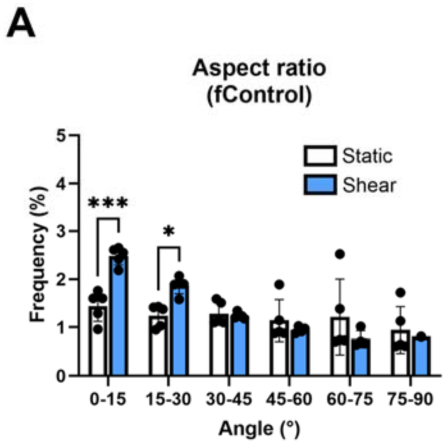

Fig. S3 Aspect ratio quantification of BECs exposed to static and shear flow conditions.

BEC alignment was quantified in iPSC-BECs under shear or static conditions. Average aspect ratio of all cells within the field of view were quantified for a minimum of five images using ImageJ using immunocytochemistry images stained for GLUT1 to identify cell borders.

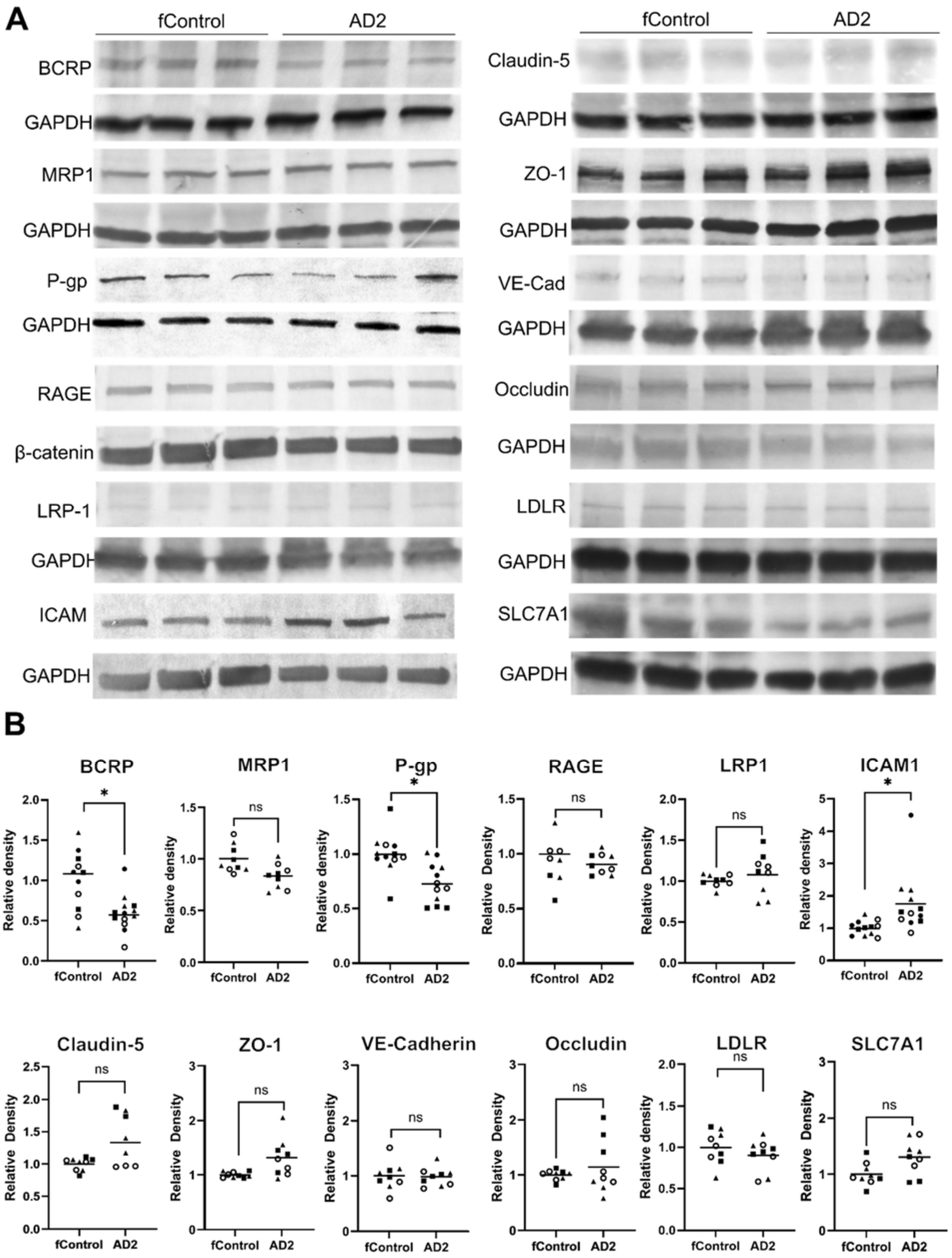
A
fControl
AD2
BCRP
GAPDH
MRP1
P-gp
RAGE
β-catenin
LRP-1
ICAM
Claudin-5
ZO-1
VE-Cad
Occludin
LDLR
SLC7A1
B
BCRP
MRP1
P-gp
RAGE
LRP1
ICAM1
Claudin-5
ZO-1
VE-Cadherin
Occludin
LDLR
SLC7A1
Relative density
Relative Density
ns
*
fControl
AD2

Figure S4. Western blot gel images used for densitometry analysis. A) Gel images used for densitometry analysis using three technical replicates for each cell line. Corresponding loading control bands are shown below each marker of interest. B) Results of densitometry analysis reported as relative density of AD2-BEC bands normalized to fControl-BEC bands. AD2-BECs showed a reduction in BCRP ($p = 0.0372$) and P-gp ($p = 0.0203$) and an increase in ICAM-1 expression ($p = 0.0392$).

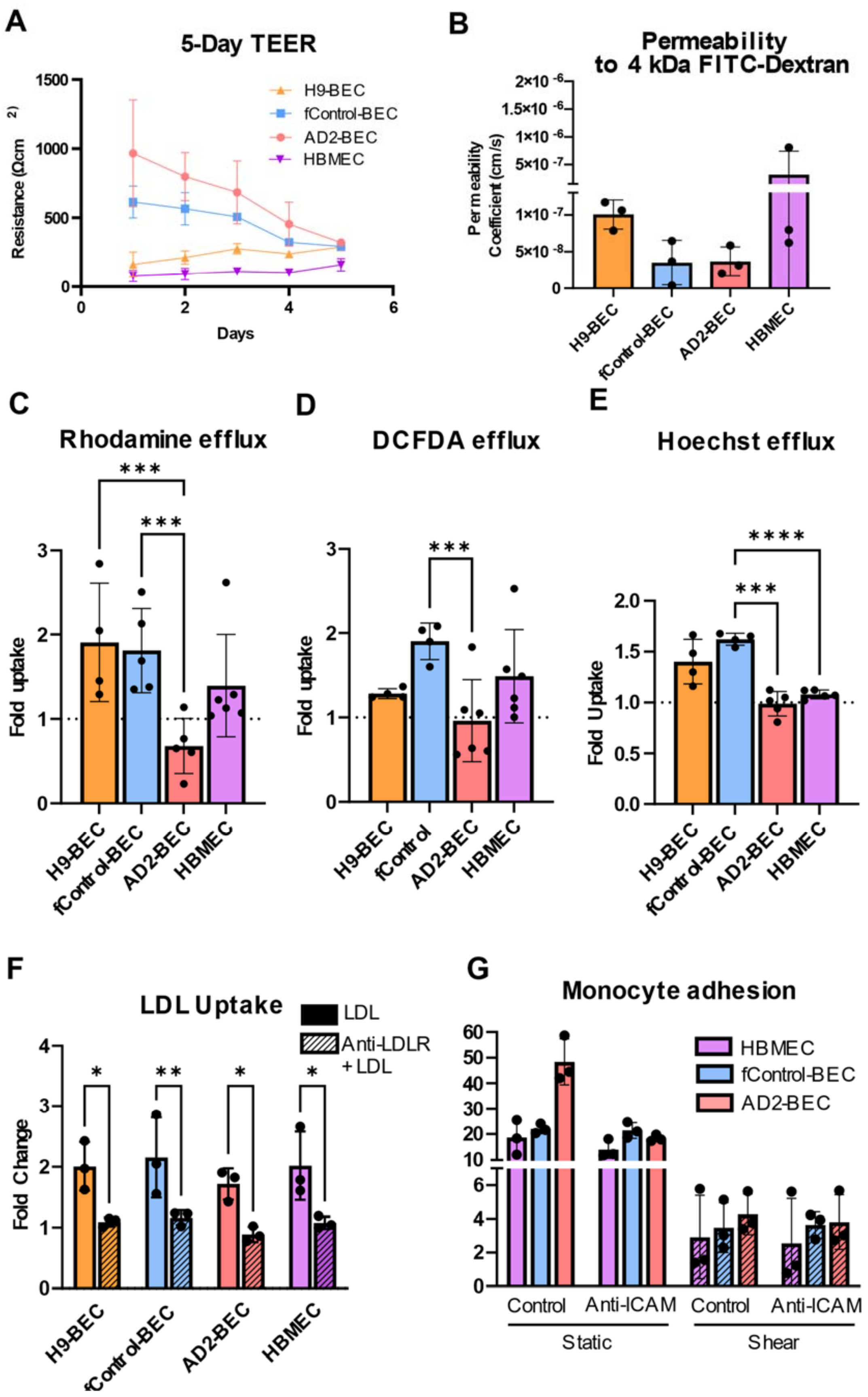
A
5-Day TEER
Resistance (Ωcm 2)
Days
H9-BEC
fControl-BEC
AD2-BEC
HBMEC
B
Permeability
to 4 kDa FITC-Dextran
Permeability
Coefficient (cm/s)
C
Rhodamine efflux
Fold uptake
D
DCFDA efflux
Fold uptake
E
Hoechst efflux
Fold Uptake
F
LDL Uptake
LDL
Anti-LDLR + LDL
Fold Change
G
Monocyte adhesion
Control
Anti-ICAM
Static
Shear

Figure S5. Characterization of immortalized HBMECs for comparison with differentiated brain endothelial-like cells. To ensure iPSC and hESC-differentiated BECs control lines were appropriate, select assays were performed using an immortalized human BEC line (HBMEC) including A) 5-day TEER B) permeability to 4 kDa FITC-dextran, C) rhodamine efflux transport, D) DCFDA efflux transport, E) Hoechst efflux transport, F) LDL-uptake, and G) monocyte adhesion. In most instances, fControl-BECs, H9-BECs and HBMECs reported similar results.

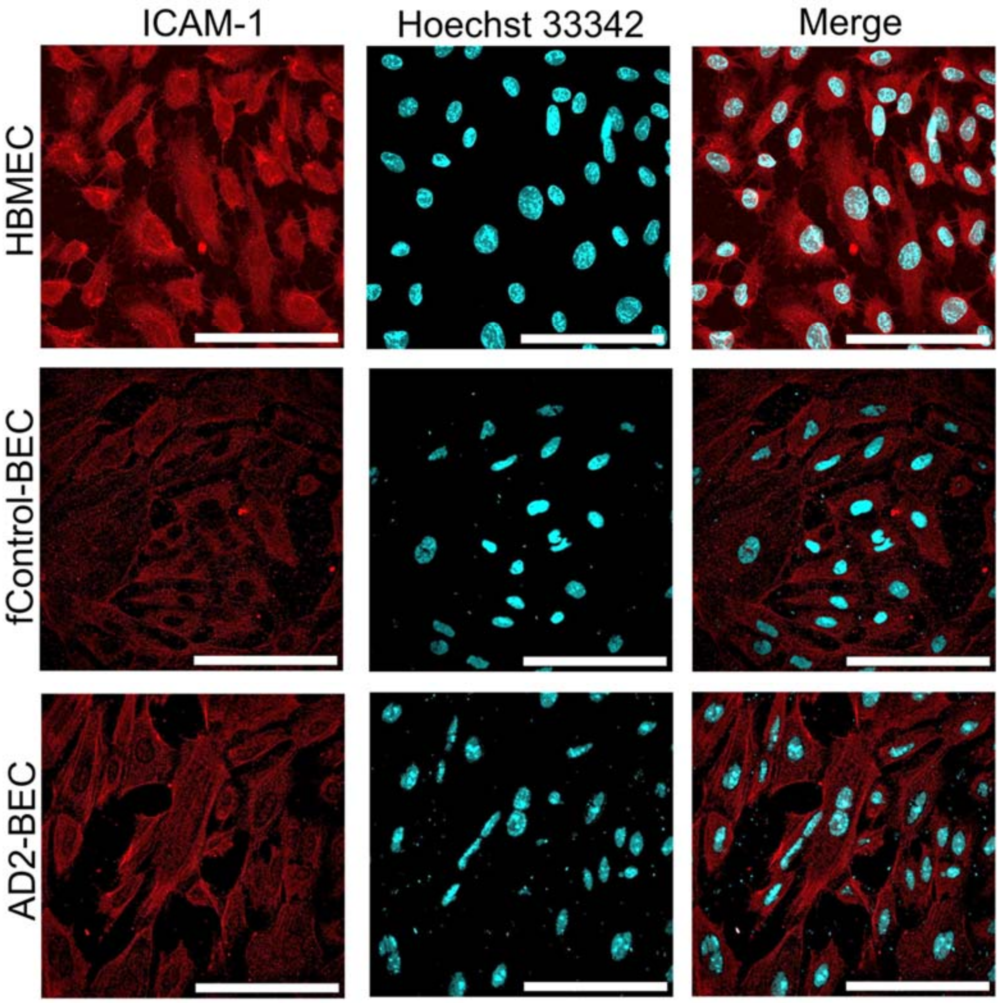

Fig S6. Representative images of ICAM-1 immunostaining on immortalized HBMECs and iPSC-derived BECs. Cells were fixed in 100% ice cold methanol and stained with anti human ICAM-1 antibody (BBA3, R&D Systems, 25 µg/mL, overnight at 4°C). All scale bars = 100 µm.

Table S1. iPSC and hESC cell line information

Cell line	Unique parental cell line ID	Lot number	Patient age at collection	Patient sex
H9 hESC	WAe009-A	WB0299		F
fControl iPSC	10-005_0949	E088-3A	33	F
AD2 iPSC	10-005_0950	E088-3E	37	F

Table S2. Staining conditions of antibodies used for western blot and immunocytochemistry

Marker	Host	Vendor	Catalogue Number	Concentration
Claudin-5	Mouse	Thermofisher Scientific	35-2500	1:1000 (WB), 1:1000 (ICC)
Occludin	Mouse	Abcam	Ab33168	1:500 (WB), 1:1000 (ICC)
VE-Cadherin	Rabbit	Abcam	Ab33168	1:1000 (WB), 1:50 (ICC)
ZO-1	Rabbit	Thermofisher Scientific	33-9100	1:500 (WB), 1:1000 (ICC)
PECAM1/CD31	Rabbit	Thermofisher Scientific	MA5-13188	1:500 (WB), 1:100 (ICC)
GLUT1	Mouse	Thermofisher Scientific	MA5-11315	1:500 (WB). 1:200 (ICC)
SLC7A1	Rabbit	Thermofisher Scientific	PA5-114947	1:1000 (WB), 1:1000 (ICC)
LDLR	Rabbit	Thermofisher Scientific	MA5-32075	1:1000 (WB), 1:1000 (ICC)
RAGE	Rabbit	Abcam	Ab37647	1:1000 (WB), 1:1000 (ICC)
LRP-1	Rabbit	Abcam	Ab214039	1:50,000 (WB), 1:10,000 (ICC)
P-gp	Mouse	Millipore Sigma	P7965	1:500 (WB), 1:1000 (ICC)
BCRP	Rabbit	Abcam	Ab207732	1:1000 (WB), 1:1000 (ICC)
MRP1	Mouse	Abcam	Ab24102	1:500 (WB), 1:1000 (ICC)
ICAM1	Mouse	Thermofisher Scientific	MA5-13021	1:100 (WB)
ICAM1	Mouse	R&D Systems	BBA3	25 µg/mL (ICC)
Anti-mouse	Goat	Thermofisher Scientific	A-11001, A-21236	1:200 (Flow), 1:2000 (ICC)
Anti-rabbit	Goat	Thermofisher Scientific	A-11008, A-21245	1:200 (Flow), 1:2000 (ICC)
Anti-mouse (HRP-conjugated)	Mouse	Cell Signalling Technology	7076	1:2000 (WB)
Anti-rabbit (HRP-conjugates)	Goat	Thermofisher Scientific	31460	1:50000 (WB)

Table S3. Efflux transport receptor inhibition assay treatment conditions

Transporter	Inhibitor	Substrate
P-gp	Cyclosporine A (10 μM)	Rhodamine 123 (10 μM)
BCRP	KO143 (1 μM)	Hoechst 33342 (10 μM)
MRP	MK571(10 μM)	H2DCFDA (10 μM)
